# Recombinant expression and downstream processing of the disulfide-rich tumor-targeting peptide chlorotoxin

**DOI:** 10.3892/etm.2013.1234

**Published:** 2013-07-24

**Authors:** XIAO-MIN WANG, XIAO LUO, ZHAN-YUN GUO

**Affiliations:** Institute of Protein Research, College of Life Sciences and Technology, Tongji University, Shanghai 200092, P.R. China

**Keywords:** chlorotoxin, disulfides, recombinant expression, purification, refolding, gliomas

## Abstract

Chlorotoxin (CTX) is a scorpion-derived disulfide-rich peptide that targets malignant tumors by binding the cell surface matrix metalloproteinase-2 and annexin A2. Various CTXs labeled with functional moieties have shown great potential for tumor diagnosis and treatment. In the present study, we established an efficient approach for preparing mature CTX that may be used for experimental and therapeutic purposes. The designed CTX precursors carried either a 6xHis-tag or a 6xHis-tag and a glutathione transferase (GST)-tag and were recombinantly expressed in *Escherichia coli*. Following S-sulfonation, the precursors were purified using immobilized metal-ion affinity chromatography. Subsequent to the removal of the tag by enterokinase cleavage and *in vitro* oxidative refolding, mature CTX was obtained with a considerable yield. The yield of mature CTX whose precursors carried a 6xHis-tag and a GST-tag (2 mg per liter of culture) was ∼10-fold that of the mature CTX whose precursors carried a 6xHis-tag (150–200 *μ*g per liter of culture). The folded CTX inhibited the migration of glioma cells in a concentration-dependent manner, suggesting it was biologically active.

## Introduction

Chlorotoxin (CTX) is a scorpion-derived bioactive peptide with 36 amino acids and four disulfide bonds. It was first isolated from the venom of *Leiurus quinquestriatus* in 1993 and was named chlorotoxin due to its ability to block small-conductance chloride channels (1). Following its discovery, CTX was observed to preferentially bind to malignant gliomas and tumors of neuroectodermal origin (2,3). Cell surface matrix metalloproteinase-2 (4) and annexin A2 (5) have been identified as the molecular targets of CTX on the tumor cells. Radionuclide iodine-131-labeled, chemically synthesized CTX (commercial name ^131^I-TM601) has been designated as an orphan drug for the treatment of malignant gliomas and melanomas by the US Food and Drug Administration (FDA). Other fluorescent dye-labeled and nanoparticle-labeled CTXs have also shown great potential for the diagnosis and treatment of malignant tumors (6–14).

Due to its small size, CTX is a promising tumor-targeting peptide and significant quantities of mature CTX are needed for therapeutic and experimental purposes. In the present study, we established an efficient approach for the preparation of mature CTX through the recombinant expression of designed CTX precursors in *Escherichia coli* (*E. coli)* and subsequent *in vitro* enzymatic and oxidative refolding.

## Materials and methods

### Materials

The oligonucleotide primers were chemically synthesized at Biosune Biotechnology Co. Ltd. (Shanghai, China) and enterokinase was purchased from New England Biolabs, Inc. (Ipswich, MA, USA). Agilent reverse-phase columns (Agilent Technologies, Santa Clara, CA, USA), including an analytical column (Zorbax 300SB-C18; 4.6×250 mm) and a semi-preparative column (Zorbax 300SB-C18; 9.4×250 mm) were used in the experiments. The peptide was eluted from the columns with an acetonitrile gradient composed of solvent A and solvent B. Solvent A was 0.1% aqueous trifluoroacetic acid (TFA), and solvent B was acetonitrile containing 0.1% TFA. The elution gradient was as follows: 0 min, 10% solvent B; 3 min, 10% solvent B; 53 min, 60% solvent B; 55 min, 100% solvent B; 56 min, 100% solvent B and 57 min, 10% solvent B. The flow rate for the analytical column was 0.5 ml/min, while that for the semi-preparative column was 1.0 ml/min. The eluted peptide was detected by UV absorbance at 280 and 214 nm.

### Gene construction, recombinant expression and purification of 6xHis-CTX

The gene of 6xHis-CTX was constructed from two chemically synthesized DNA primers. Subsequent to annealing, elongation by T4 DNA polymerase and cleavage by the restriction enzymes *Nde*I and *Eco*RI, the DNA fragment was ligated into a pET vector pretreated with the same restriction enzymes. Its sequence was confirmed using DNA sequencing. The expression construct pET/6xHis-CTX was then transformed into the *E. coli* strain BL21 Star™ (DE3), prior to the transformed cells being cultured in liquid Luria-Bertani (LB) medium, containing 10 g/l tryptone, 5 g/l yeast extract, 10 g/l NaCl medium (with 100 *μ*g/ml ampicillin), to an optical density at 600 nm (OD_600_) of 1.0 at 37°C, with vigorous shaking (250 rpm). Following induction by 1.0 mM isopropyl thio-β-D-galactoside (IPTG) at 37°C for 6–8 h, the *E. coli* cells were harvested by centrifugation (5,000 × g, 10 min), resuspended in lysis buffer (50 mM Tris-HCl, pH 8.5; 0.5 M NaCl) and lysed using sonication. Subsequent to further centrifugation (10,000 × g, 15 min), the inclusion body pellet was resuspended in solubilizing buffer (50 mM Tris-HCl, 6 M guanidine chloride; pH 8.5) and S-sulfonated by the addition of solid sodium sulfite and sodium tetrathionate to the final concentrations of 200 mM and 150 mM, respectively. The S-sulfonation reaction was carried out at 4°C with gentle shaking for 2–3 h. Following centrifugation (10,000 × g, 15 min), the supernatant was loaded onto an Ni^2+^ column that was pre-equilibrated with the washing buffer (50 mM Tris-HCl, 3 M guanidine chloride; pH 8.5). The S-sulfonated precursor was then eluted from the column by a step-wise increase of imidazole concentration in the washing buffer. The eluted S-sulfonated 6xHis-CTX was subsequently further purified using C18 reverse-phase high-performance liquid chromatography (HPLC) and lyophilized. The molecular mass was measured using electro-spray mass spectrometry.

### Gene construction, recombinant expression and purification of glutathione transferase (GST)-6xHis-CTX

The coding region of 6xHis-CTX was amplified by polymerase chain reaction (PCR) using pET/6xHis-CTX as the template. The amplified DNA fragment was digested by the restriction enzymes *Bam*HI and *Xho*I and subsequently ligated into a pGEM-4T-1 vector, providing the GST tag, pretreated with the same restriction enzymes. Its sequence was confirmed by DNA sequencing. Following this, the construct pGEM/GST-6xHis-CTX was transformed into the *E. coli* strain BL21 Star™ (DE3) and the transformed cells were cultured in liquid terrific broth (TB) medium, containing 12 g/l tryptone, 24 g/l yeast extract, 0.4% (V/V) glycerol, 17 mM KH_2_PO_4_, 72 mM K_2_HPO_4_;(with 100 *μ*g/ml ampicillin), to an OD_600_ of ∼5.0 at 37°C, with vigorous shaking (250 rpm). Subsequent to overnight induction with 1.0 mM IPTG at 37°C, the *E. coli* cells were harvested by centrifugation (5,000 × g, 10 min), resuspended in lysis buffer (50 mM phosphate, pH 7.4; 0.5 M NaCl) and lysed using a French press. The soluble GST-6xHis-CTX in the supernatant was then subjected to S-sulfonation by the addition of solid sodium sulfite and sodium tetrathionate to final concentrations of 200 and 150 mM, respectively. Following shaking at 4°C for 2–3 h, the S-sulfonated sample was loaded onto an Ni^2+^ column that was pre-equilibrated with the washing buffer (50 mM Tris, 150 mM NaCl; pH 8.0). The S-sulfonated GST-6xHis-CTX was eluted from the column by a step-wise increase of imidazole concentration in the washing buffer. The eluted GST-6xHis-CTX fraction was concentrated by ultrafiltration for enzymatic digestion in the next step.

### Enterokinase cleavage of the S-sulfonated CTX precursors and in vitro refolding

The S-sulfonated CTX precursors (6xHis-CTX and GST-6xHis-CTX) were digested by enterokinase (peptide:enzyme molar ratio 10^5^:1) in the digestion buffer (10 mM Tris-HCl, 50 mM NaCl, 10 mM CaCl_2_; pH 8.0) at 25°C overnight. For 6xHis-CTX, the digestion mixture was directly subjected to *in vitro* refolding. For GST-6xHis-CTX, the digestion mixture was first subjected to gel filtration (Sephadex G-50 column, Sinopharm Chemical Reagent Co., Ltd., China) and the sulfonated CTX fraction was collected and used for refolding. For oxidative refolding, the S-sulfonated CTX was initially treated with 10 mM dithiothreitol (DTT) at room temperature for 15 min, prior to being 10-fold diluted into the pre-incubated refolding buffer (0.5 M L-arginine, 1.0 mM EDTA and 2.0 mM oxidized glutathione; pH 8.5). The refolding reaction was carried out at 4°C for 1–2 h. Following this, the refolding mixture was acidified to pH 3.0 using TFA and subjected to a C18 reverse-phase HPLC. The eluted, refolded CTX fraction was manually collected, lyophilized and analyzed using mass spectrometry.

### Activity assay of the folded CTX

The activity of the recombinant CTX was evaluated using a tumor cell invasion assay in matrigel. U251-MG cells (Cell Bank in Shanghai Institutes For Biological Sciences, CAS, China) were cultured in Dulbecco’s modified Eagle’s medium (DMEM) supplemented with 10% fetal bovine serum and antibiotics. Following trypsin digestion, the cells were washed with phosphate-buffered saline (PBS), resuspended in DMEM without fetal bovine serum and seeded into the matrigel-covered invasion chamber (5×10^4^ cells/chamber, 8-*μ*m pores; BD Biosciences, Franklin Lakes, NJ, USA). The chambers were then placed in 24-well plates containing DMEM with 10% fetal bovine serum. The cells were cultured at 37°C for 1 h, prior to indicated concentrations of CTX being added into the chamber. The cells were subsequently continuously cultured at 37°C for 20 h. Following this, the cells at the inner side of the invasion chamber were scraped off and the migrated cells at the outside of the chamber were stained with crystal violet and counted under a microscope.

### Statistical analysis

Sigmaplot 12.1 from Systat Software Inc. (http://www.sigmaplot.com/) was used for statistical analysis. A t-test was used to analyze statistically significant differences.

## Results

### Recombinant expression and purification of the designed CTX precursors

**Figure 1.**
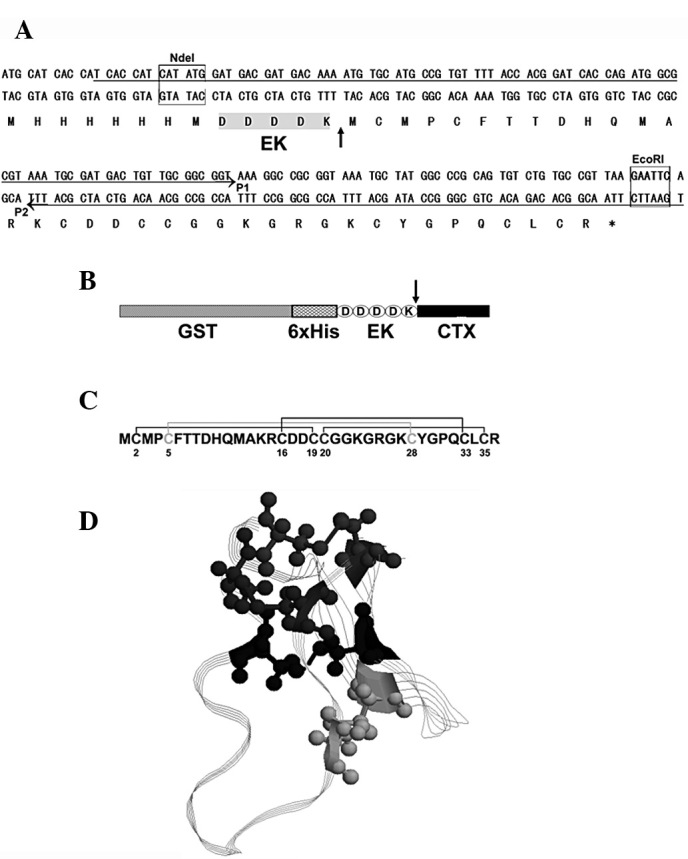
(A) Nucleotide and amino acid sequence of the designed 6xHis-chlorotoxin (CTX) precursor. The two primers used to construct the gene of the precursor are underlined and labeled. The enterokinase (EK) cleavage site (DDDDK) is shaded. The asterisk indicates the stop codon. (B) Cartoon presentation of the glutathione transferase (GST)-6xHis-CTX precursor. (C) Amino acid sequence and disulfide linkages of mature CTX. (D) The previously revealed nuclear magnetic resonance (NMR) structure of CTX (Protein Data Bank ID: 1CHL).

To prepare mature CTX using heterologous expression in *E. coli*, a 6xHis-CTX precursor was designed, as shown in Fig. 1A. A 6xHis-tag, to facilitate purification, was present at the precursor N-terminal. An enterokinase cleavage site (DDDDK) was introduced between the 6xHis-tag and the mature CTX to enable the removal of the tag following purification. To improve its expression level, *E. coli*-biased codons were used in the synthetic gene. A GST-6xHis-CTX precursor was also constructed, as shown in Fig. 1B. In this precursor, a large GST-tag was fused at the N-terminal in order to stabilize the small CTX, which was prone to degradation in *E. coli*.

**Figure 2.**
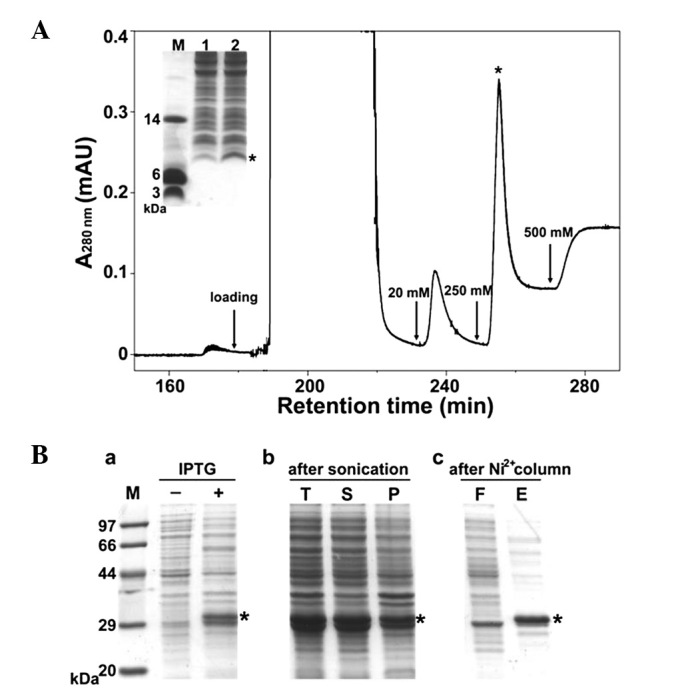
(A) Purification of the S-sulfonated 6xHis-chlorotoxin (CTX) precursor using immobilized metal ion affinity chromatography. The peak of the S-sulfonated precursor is indicated by an asterisk. Inner panel, tricine sodium dodecyl sulfate-polyacrylamide gel electrophoresis (SDS-PAGE) analysis of the 6xHis-CTX expression *in E. coli*. M, marker; Lane 1, prior to isopropyl thio-β-D-galactoside (IPTG) induction; Lane 2, following IPTG induction. The band of 6xHis-CTX is indicated by an asterisk. (B) SDS-PAGE analyses of the glutathione transferase (GST)-6xHis-CTX precursor at different purification stages. (Ba) SDS-PAGE analysis of GST-6xHis-CTX expression: (−) prior to, and (+) subsequent to IPTG induction. (Bb) SDS-PAGE analysis of the solubility of GST-6xHis-CTX. T, total lysate; S, supernatant; P, pellet. (Bc) SDS-PAGE analysis of Ni^2+^ column-purified GST-6xHis-CTX. The band of GST-6xHis-CTX is indicated by an asterisk. F, flow-through; E, eluted fraction by 100 mM imidazole.

6xHis-CTX was recombinantly expressed in the *E. coli* strain BL21 Star™ (DE3) under IPTG induction. As analyzed using tricine sodium dodecyl sulfate-polyacrylamide gel electrophoresis (SDS-PAGE), a band with a molecular weight of ∼8 kDa was significantly increased subsequent to induction (Fig. 2A, inner panel). Once the *E. coli* cells had been lysed by sonication, the precursor was shown to be present in the pellet due to the formation of inclusion bodies (data not shown). The precursor was solubilized by guanidine chloride and S-sulfonated, which broke the inter-chain disulfide cross-linking and reversibly modified the eight cysteine residues of the precursor with negatively charged sulfonate moieties. Our later experiments showed that the precursor refolded into a mixture of disulfide isomers due to the presence of the tag; therefore, the removal of the 6xHis-tag was a prerequisite for the efficient oxidative folding of the recombinant CTX. Furthermore, the enzyme enterokinase was not able not cleave the CTX precursor with disulfide bonds due to steric hindrance; thus, S-sulfonation was a necessary step for efficient enzyme cleavage and oxidative refolding. The S-sulfonated precursor was then purified using an immobilized metal-ion affinity chromatography (Ni^2+^ column), as shown in Fig. 2A. The eluted precursor fraction (indicated by an asterisk) was further purified using C18 reverse-phase HPLC.

GST-6xHis-CTX was also heterologously expressed in the *E. coli* strain BL21 Star™ (DE3). As analyzed using SDS-PAGE, a 30 kDa band that was consistent with the expected molecular weight of the precursor was significantly increased following IPTG induction (Fig. 2Ba). Once the *E. coli* cells had been lysed using a French press, ∼60% of the precursor was present in the supernatant (Fig. 2Bb). The soluble GST-6xHis-CTX was then subjected to S-sulfonation and purified using immobilized metal ion affinity chromatography (Ni^2+^ column). The S-sulfonation step was necessary for the efficient enterokinase cleavage of the precursor. As analyzed using SDS-PAGE (Fig. 2Bc), the S-sulfonated GST-6xHis-CTX was eluted from the Ni^2+^ column by 100 mM imidazole. The eluted precursor fraction was subjected to ultrafiltration in order to concentrate the precursor and partially remove the salt.

### Enterokinase cleavage and in vitro refolding

**Figure 3.**
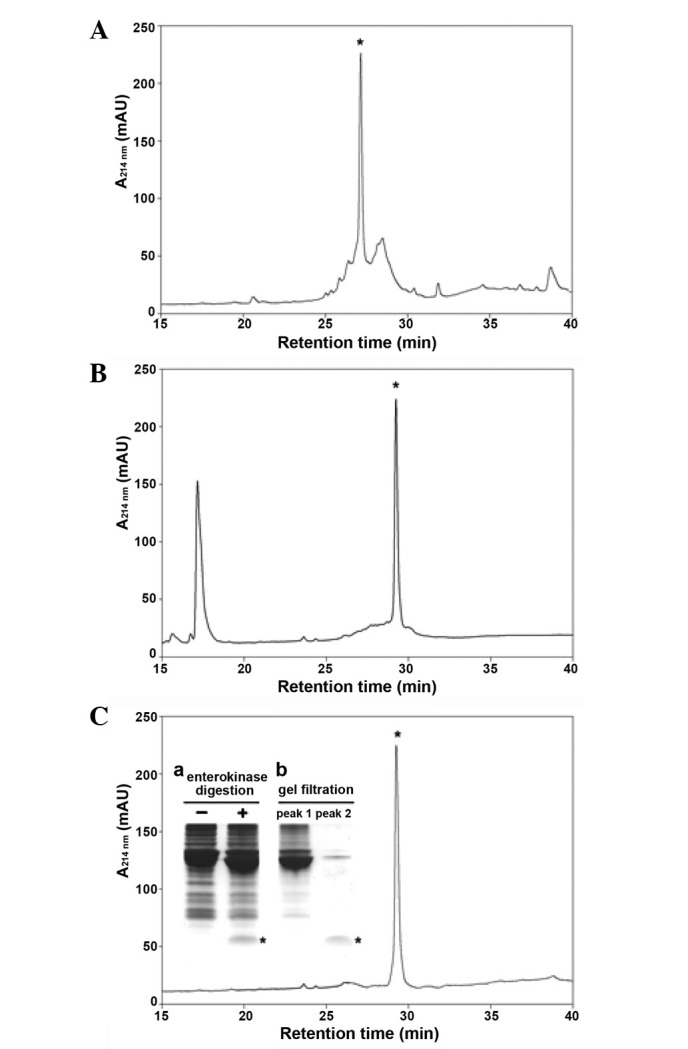
Enterokinase cleavage of CTX precursors and *in vitro* refolding of the S-sulfonated chlorotoxin (CTX). (A) High-performance liquid chromatography (HPLC) profile of the S-sulfonated 6xHis-CTX eluted from the Ni^2+^ column. The peak of S-sulfonated 6xHis-CTX is indicated by an asterisk. (B) HPLC analysis of the CTX refolding mixture derived from S-sulfonated 6xHis-CTX. The peak of the mature CTX is indicated by an asterisk. (C) HPLC analysis of the refolded CTX derived from glutathione transferase (GST)-6xHis-CTX. The peak of the mature CTX is indicated by an asterisk. Inner panel (a) tricine sodium dodecyl sulfate-polyacrylamide gel electrophoresis (SDS-PAGE) analysis of enterokinase digestion of the S-sulfonated GST-6xHis-CTX: (−) prior to, and (+) subsequent to digestion. Inner panel (b) tricine SDS-PAGE analysis of the peaks eluted from gel filtration. The band of the S-sulfonated CTX is indicated by an asterisk.

The S-sulfonated 6xHis-CTX precursor, eluted from the Ni^2+^ column, was analyzed using C18 reverse-phase HPLC, as shown in Fig. 3A. The measured molecular mass of the eluted peak (indicated by an asterisk) was 6,318.0 Da, which was consistent with the expected value (6,319.0 Da) of the S-sulfonated precursor. The purified S-sulfonated precursor was then subjected to sequential enterokinase digestion and *in vitro* refolding. As analyzed using HPLC (Fig. 3B), two major peaks appeared. The first peak had a measured molecular mass of 1,692.0 Da, which was consistent with the expected value (1,691.8 Da) of the N-terminal 6xHis-tag. The second peak had a measured molecular mass of 3,996.0 Da, which was consistent with the expected value (3,996.8 Da) of mature CTX. The final yield of mature CTX was 150–200 *μ*g per liter of culture.

The S-sulfonated GST-6xHis-CTX was also subjected to enterokinase digestion in order to remove the GST-tag and 6xHis-tag. As shown in Fig. 3C (inner panel, a), a band with the expected molecular weight of the S-sulfonated CTX (indicated by an asterisk) appeared following enterokinase digestion. The digestion mixture was then subjected to gel filtration and two peaks were eluted from the Sephadex G-50 cloumn (data not shown). As analyzed using tricine SDS-PAGE (Fig. 3C, inner panel b), the second eluted peak was the S-sulfonated CTX fraction. Subsequent to refolding, a major peak appeared on the HPLC (Fig. 3C) with a measured molecular mass (3,998.0 Da) that was consistent with the theoretical value (3,996.8 Da) of the mature folded CTX. The final yield of mature CTX was 2 mg per liter of culture.

### Activity assay of the recombinant CTX

**Figure 4.**
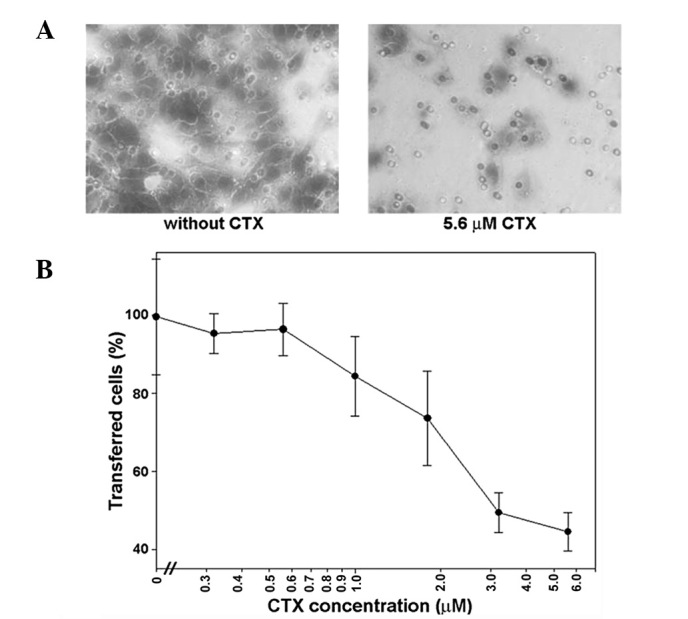
Inhibition of cultured glioma U251-MG cell invasion by the recombinant chlorotoxin (CTX). (A) Representative microscopic image of the cells migrating through the matrigel with or without CTX. The migrated cells were stained using crystal violet. Magnification, ×200. (B) Concentration-dependent inhibition of U251-MG cell invasion by recombinant CTX.

The activity of the recombinant CTX was assessed by evaluating its ability to inhibit cell invasion in matrigel. As shown in Fig. 4A, the glioma-derived U251-MG cell migration through the matrigel was significantly inhibited by 5.6 *μ*M mature CTX. This inhibitory effect was concentration-dependent. The maximum inhibition reached ∼60%, with an IC_50_ value of ∼1.5 *μ*M (Fig. 4B), which suggested that the recombinant CTX was biologically active.

## Discussion

CTX is a disulfide-rich peptide with 36 amino acids and four disulfide bonds. Although CTX may be prepared through chemical synthesis and subsequent oxidative folding, the final yield of this process is insufficient (15,16). Therefore, the present study attempted to prepare CTX using recombinant expression in *E. coli*. To facilitate purification and improve the expression level, a 6xHis-tag alone or with a large GST-tag was fused to the N-terminus of the CTX. However, it was demonstrated that the 6xHis-CTX was not able to fold into a unique disulfide isomer (data not shown), which was most likely due to the disturbance of the N-terminal extension (6xHis-tag and the enterokinase cleavage site). In addition, the folded 6xHis-CTX (a mixture of disulfide isomers) was not efficiently digested by enterokinase, most likely due to steric hindrance, since one disulfide bond was in the proximity of the cleavage site. Therefore, an S-sulfonation approach was employed, by which the eight cysteine residues of the CTX precursors were reversibly modified by sulfonate moieties. The S-sulfonated CTX precursors were highly soluble in the enzyme digestion buffer and were efficiently cleaved by enterokinase. Subsequent to the removal of the N-terminal tag, the S-sulfonated CTX was efficiently refolded *in vitro* with ∼80% yield under optimized conditions. In addition, it was demonstrated that the use of the precursor (GST-6xHis-CTX) in the expression significantly improved the final yield of mature CTX (versus that with 6xHis-CTX). Therefore, the present study provided an efficient approach for the preparation of active CTX and its analogs for further investigation.
